# The Dorsal Skinfold Chamber as a New Tympanic Membrane Wound Healing Model: Intravital Insights into the Pathophysiology of Epithelialized Wounds

**DOI:** 10.1159/000519774

**Published:** 2021-12-02

**Authors:** Daniel Strüder, Christoph Lachmann, Sara Maria van Bonn, Eberhard Grambow, Sebastian P. Schraven, Robert Mlynski, Brigitte Vollmar

**Affiliations:** ^a^Department of Otorhinolaryngology, Head and Neck Surgery “Otto Körner”, Rostock University Medical Center, Rostock, Germany; ^b^Institute for Experimental Surgery, Rostock University Medical Center, Rostock, Germany; ^c^Department of General, Visceral, Vascular and Transplantation Surgery, Rostock University Medical Center, Rostock, Germany

**Keywords:** Tympanic membrane perforation, Biomaterials, Wound dressings, Wound healing models

## Abstract

**Background:**

Tympanic membrane perforations (TMPs) are a common complication of trauma and infection. Persisting perforations result from the unique location of the tympanic membrane. The wound is surrounded by air of the middle ear and the external auditory canal. The inadequate wound bed, growth factor, and blood supply lead to circular epithelialization of the perforation's edge and premature interruption of defect closure. Orthotopic animal models use mechanical or chemical tympanic membrane laceration to identify bioactive wound dressings and overcome premature epithelialization. However, all orthotopic models essentially lack repetitive visualization of the biomaterial-wound interface. Therefore, recent progress in 3D printing of customized wound dressings has not yet been transferred to the unique wound setup of the TMP. Here, we present a novel application for the mice dorsal skinfold chamber (DSC) with an epithelialized full-thickness defect as TMP model.

**Methods:**

A circular 2-mm defect was cut into the extended dorsal skinfold using a biopsy punch. The skinfold was either perforated through both skin layers without prior preparation or perforated on 1 side, following resection of the opposing skin layer. In both groups, the wound was sealed with a coverslip or left unclosed (*n* = 4). All animals were examined for epithelialization of the edge (histology), size of the perforation (planimetry), neovascularization (repetitive intravital fluorescence microscopy), and inflammation (immunohistology).

**Results:**

The edge of the perforation was overgrown by the cornified squamous epithelium in all pre­parations. Reduction in the perforation's size was enhanced by application of a coverslip. Microsurgical preparation before biopsy punch perforation and sealing with a coverslip enabled repetitive high-quality intravital fluorescence microscopy. However, spontaneous reduction of the perforation occurred frequently. Therefore, the direct biopsy punch perforation without microsurgical preparation was favorable: spontaneous reduction did not occur throughout 21 days. Moreover, the visualization of the neovascularization was sufficient in intravital microscopy.

**Conclusions:**

The DSC full-thickness defect is a valuable supplement to orthotopic TMP models. Repetitive intravital microscopy of the epithelialized edge enables investigation of the underlying pathophysiology during the transition from the inflammation to the proliferation phase of wound healing. Using established analysis procedures, the present model provides an effective platform for the screening of bioactive materials and transferring progress in tissue engineering to the special conditions of tympanic membrane wound healing.

## Introduction

Tympanic membrane perforations (TMPs) are a common complication of trauma, ear-tubes, and otitis media. Despite spontaneous closure in 38–94%, 30 million patients worldwide suffer from hearing loss, otorrhea, and recurrent otitis media because of chronic TMPs[1, 2, 3, 4, 5, 6, 7, 8]. Routine therapy of acute TMPs comprises chemical debridement (e.g., policresulen) and splinting with inert silicone splints, gelatin sponges, paper strips, or egg shell membranes [8, 9, 10, 11, 12]. However, previous research failed to show robust effects of tympanic membrane splinting using inert materials [7, 13, 14, 15, 16]. For example, silicone splinting was successful in 51.6% (*n* = 128), while spontaneous closure occurred in 53.3% of the control group (*n* = 30) [7]. Persisting perforations cause chronic middle ear infections and require microscopic tympanoplasty with cartilage or fascia autografting. The success rate of 43–100% depends on the surgeon's experience and the surgical technique [1, 2, 3, 6, 7].

**Fig. 1:**
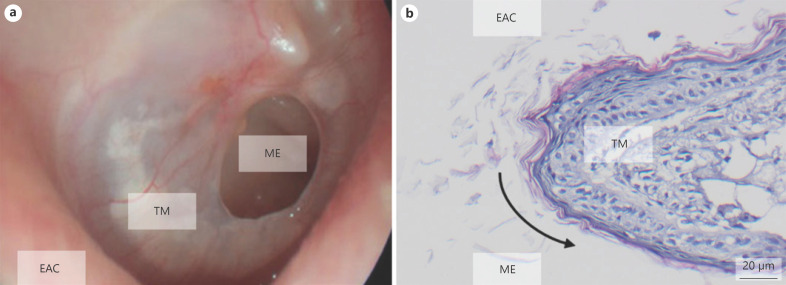
Epithelialization of TMPs. **a** Ear microscopy of a TMP shows round epithelialized margins in the TMP. **b** Histology (H&E) of a TMP confirms epithelialization of the defect. The black arrow illustrates the overgrowth of the keratinizing squamous epithelium over the defect margin. EAC, external auditory canal; ME, middle ear; TM, tympanic membrane; TMP, tympanic membrane perforation.

Pathological tympanic membrane wound healing results from the unique location. The wound is surrounded by the air of the middle ear and the external auditory channel. The missing wound bed impedes cell migration, perfusion, and growth factor supply [2, 3, 17, 18]. The essential wound healing phases − inflammation and proliferation − are abbreviated. Therefore, the circular edge of the perforation epithelializes and interrupts defect closure (shown in Fig. 1) [2, 13, 18, 19, 20, 21, 22].

Modern wound dressings with specific microarchitecture (pore size and elasticity) and biology (cell adhesion/migration and selective drug release) support cell migration, granulation tissue formation, and growth factor supply. Recent research showed promising tympanic membrane regeneration using growth factor-, stem cell-, and antibiotic-loaded electrospun fibers; polylactic acid; chitosan; and alginate scaffolds [1, 13, 19, 20, 23, 24, 25, 26, 27, 28, 29, 30, 31, 32, 33]. However, in vivo experiments were not standardized, and most scaffolds released the active substances with a single burst. Sequential drug release could be more effective in overcoming the specific pathology of premature epithelialization during the inflammation-proliferation transition. Extension of the inflammatory phase by pro-inflammatory cytokines may induce secretion and wound bed formation. Subsequently, granulation tissue formation and proliferation may be supported by growth factor release.

However, current in vitro and in vivo tympanic membrane models cannot repetitively visualize effects on specific wound healing phases. Orthotopic TMP models have 2 major limitations: lack of wound visualization after biomaterial application and non-standardized spontaneous defect closure. Laceration of rodent tympanic membranes leads to regular spontaneous closure within weeks [34, 35, 36, 37]. Therefore, various methods of mechanical and biochemical attenuation of spontaneous closure have been reported (e.g., thermal myringotomies, epithelialized flaps, and topical mitomycin/corticoid) [3, 18, 32, 38, 39, 40, 41, 42]. Still, the resulting defect size is not standardized, and wound attenuation may interfere with wound treatment.

The second major limitation of orthotopic models is the lack of wound visualization. The low diameter of the external ear canal impedes wound observation after biomaterial application. Thus, each measurement of the defect requires removal of the biomaterial. Therefore, repetitive intravital visualization of the dynamic wound-biomaterial interface cannot be performed: *in vivo* assessment of microvascular parameters is not possible and analysis is based on (immuno-)histology at a single postmortem time [43, 44, 45, 46]. Additionally, most orthotopic models require animals with limited availability (chinchillas and guinea pigs), microsurgery (within the tight external ear canal), and housing/surveillance for 8 weeks after tympanic membrane laceration. Table 1 summarizes recent research on orthotopic models and illustrates the heterogeneity regarding animals, methods, and spontaneous closure rates between 0 and 100%. These limitations decrease reproducibility of orthotopic TMP models and restrict expedient studies of complex biomaterials. Yet, future improvements require profound knowledge about pathophysiology in the specific phases of wound healing [18].

The most elaborate model for repetitive intravital examination of wound healing and the biomaterial-wound interface is the dorsal skinfold chamber (DSC) [26, 47, 48, 49, 50, 51, 52, 53, 54, 55, 56, 57, 58, 59, 60]. Here, we present a novel application for the DSC with an epithelialized and circular full-thickness defect as a preclinical model of a TMP.

The aim of the study was the identification of a preparation with a stable size of the perforation and low angiogenesis at the wound boundary [17, 47, 61, 62, 63, 64]. This study tested 4 different preparations regarding wound epithelialization, spontaneous closure, and intravital microscopy. Using established analysis procedures, the alternative model enables the straightforward transfer of progress in tissue engineering and biomaterials to the special conditions in wound healing of the tympanic membrane.

## Methods/Design

### Animals and Ethics Statement

All in vivo experiments were conducted in accordance with the German legislation on protection of animals and the NIH Guide for the Care and Use of Laboratory Animals (Institute of Laboratory Animal Resources, National Research Council). Male hairless SKH1-hr mice (6–10 weeks of age and weight of 25–30 g) were used for all experiments. The animals were housed individually in a specific pathogen-free facility with a 12-h light-dark cycle and access to standard laboratory chow and water ad libitum.

### Study Design

**Fig. 2:**
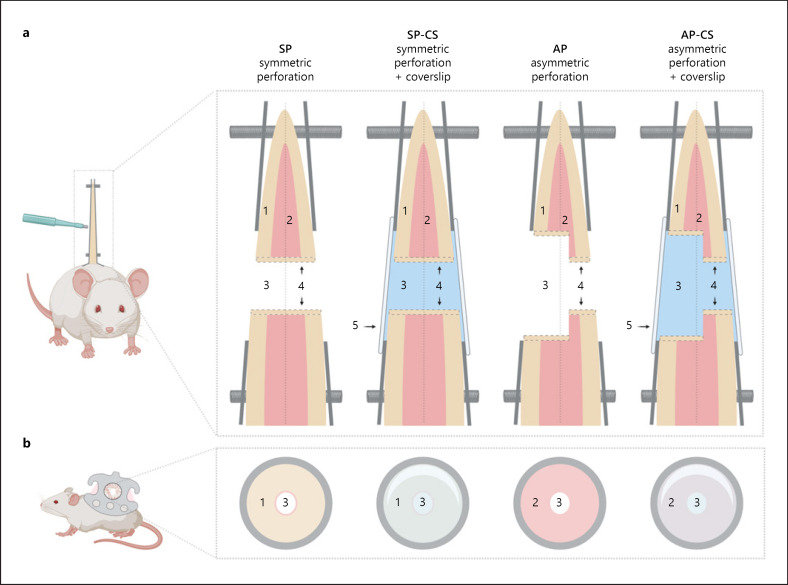
DSC preparation and experimental groups. SPs were prepared through both layers of skin (1) and panniculus carnosus muscle (2) without prior preparation of the panniculus carnosus muscle. The defect remained open or sealed a coverslip (5). The asymmetrical preparation included microsurgical exposure of the panniculus carnosus muscle (2), before excision of the full-thickness defect (3). Likewise, the chamber remained open or sealed with a coverslip (5). The edges (4) of the full-thickness perforation (3) epithelialized during wound healing (*n* = 5, transverse plane **a**, sagittal plane **b**). SP, symmetric perforation; SP-CS, symmetric perforation with coverslip; AP, asymmetric perforation; AP-CS, asymmetric perforation with coverslip; DSC, dorsal skinfold chamber.

Twenty mice were randomly allocated to 4 experimental groups: symmetric perforation without coverslip (SP), symmetric perforation with coverslip (SP-CS), asymmetric perforation with coverslip (AP-CS) and asymmetric perforation without coverslip (AP) (*n* = 5) (shown in Fig. 2). Each animal was an experimental unit and examined independently using planimetry and intravital microscopy at days 4, 8, 12, 16 and 20 after DSC preparation. Following intravital microscopy at day 20, mice were sacrificed and the tissue surrounding the perforation was saved for subsequent histology.

### Experimental Procedures

DSC modification: Mice were anesthetized by an intraperitoneal injection of ketamine/xylazine (90/25 mg/kg bw) and a heating pad (37.8°C). Microsurgery for DSC implantation has been described in detail [65, 66, 67, 68]. The significant change in the proposed model is the preparation of a standardized full-thickness defect using a 2-mm skin biopsy punch. The model was tested in 4 distinct DSC preparations: SP, SP-CS, AP, and AP-CS (shown in Fig. 2).

In the SP and SP-CS groups, the full-thickness dermal perforation was excised right after fixation of the dorsal skinfold in the titanium frame (without further preparation). The chamber remained open (SP) or sealed with coverslips from both sides and filled with saline (0.9%) (SP-CS). In the AP and AP-CS groups 1 skin layer (epidermis, subcutis, and panniculus carnosus muscle) of the extended skinfold was removed. Then, the opposing layer of subcutaneous tissue was excised microsurgically down to the opposing panniculus carnosus muscle, as described for standard DSC models [25, 26, 62, 63, 65, 67]. Then, the remaining intact layer of the skin was perforated using the biopsy punch. Again, the chamber remained open (AP) or filled with saline (0.9%) and sealed with coverslips from both sides (AP-CS). Mice were recovered for 4 days after surgery to minimize the effect of the surgical trauma.

### Intravital Microscopy

**Fig. 3:**
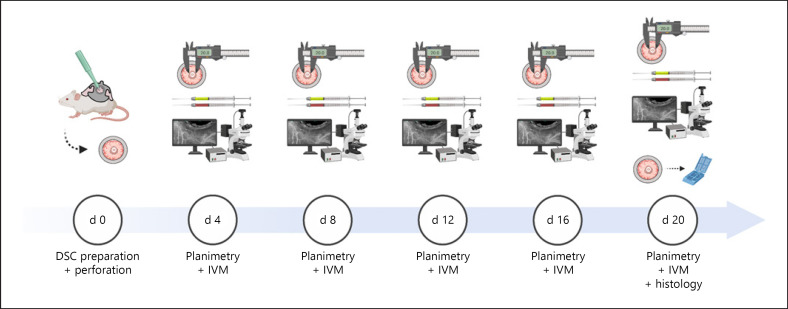
Study design and experimental procedures. The DSC was prepared on day 0 and a circular full-thickness defect was created using a 2-mm biopsy punch (d 0). After regeneration from the surgical trauma, planimetry, and IVM were performed repetitively (d 4, d 8, d 12, d 16, and d 20). During IVM, the microvascular system and leucocytes were visualized by simultaneous intravenous injection of FITC-dextran and Rhodamine 6G. Following IVM on day 20, the chamber tissue was saved for histology. IVM, intravital fluorescence microscopy; d, day; DSC, dorsal skinfold chamber; FITC, fluorescein isothiocyanate-labeled.

Repetitive planimetry and intravital microscopy were performed on days 4, 8, 12, 16, and 20 (shown in Fig. 3). Mice were anesthetized and placed on a plexiglass pad with integrated heating. First, planimetry of the perforation was performed using a stereomicroscope (IC-A; Leica Microsystems GmbH, Wetzlar, Germany). For the visualization of the microvascular system fluorescein isothiocyanate-labeled-dextran (0.05 mL, 5%, MW: 150 kD) and Rhodamine 6G (0.05 mL, 2%, MW: 496 D) were injected into the lateral tail vein (or into the retrobulbar venous plexus if tail vein injection failed). Intravital microscopy was performed with 50-, 100-, and 200-fold magnification using an Axiotech vario microscope (Carl Zeiss AG, Oberkochen, Germany) with a 100-W HBO mercury lamp with a blue filter (excitation, 450–490 nm; emission, 520 nm) and a green filter (excitation, 530–560 nm; emission, 580 nm). The microvascular parameters were examined using the blue filter and 200-fold magnification. Three regions of interest (ROIs) proximal to the perforation and their corresponding peripheral ROIs were recorded at each time point: Each time, 1 ROI in proximity to the edge of the perforation was observed for 20 s. Then, the scope was radially moved 1 field of view away from the perforation. This peripheral ROI was observed for further 20 s. Leukocyte flow dynamics and leukocyte-endothelial cell interactions were examined using the green filter and 20-fold magnification. Therefore, 3 venules in the observational window were visualized for 30 s. The microscopic images were recorded on a DVD (DMR-EX99V, Panasonic, Kadoma, Japan) using a charge-coupled video camera (FK 6990A-IQ, Pieper, Berlin, Germany) for off-line evaluation.

Computer-assisted image analysis was performed off-line using CapImage (Zeintl Software, Dreieich, Germany). The investigator was blinded for the experimental groups during off-line analysis. The size of the perforation was assessed by planimetry (mm^2^). Analysis of the microcirculation comprised vessel diameters (µm), red blood cell velocity (µm/s), and functional capillary density (mm/mm^2^). Diameters were measured perpendicularly to the vessel path. Red blood cell velocity was assessed using the line shift method. Functional capillary density was defined as the length of red blood cell-perfused capillaries per ROI. Inflammation was quantified by measurement of leukocyte number (cells/mm^2^), leukocyte flow (cells/mm^2^/s), and leukocyte-endothelium interaction (cells/mm^2^). Adherent leucocytes were defined as Rhodamine 6G-stained cells per ROI. Rolling leukocytes were defined as Rhodamine 6G-positive cells moving slower than red blood cells.

### Histology and Immunohistochemistry

Histology and immunohistochemistry were performed as previously described [65]. Briefly, the tissue was fixed in 4% phosphate-buffered formalin for 3 days and then embedded in paraffin. Sections of 4 μm were stained with hematoxylin-eosin for routine histology. Inflammation was visualized by AS-D chloroacetate esterase (CAE) leukocyte staining and F4/80 macrophage staining (polyclonal rat anti-mouse anti-F4/80, 1:10, Serotec, Hercules, United States, secondary mouse anti-rat immunoglobulin antibody, 1:200, Santa Cruz Biotechnology, Dallas, TX, USA). Ki-67 as marker for proliferating cells was labeled with a rabbit polyclonal anti-Ki-67 (1:200, Abcam, Cambridge, UK, secondary polyclonal goat anti-rabbit IgG with alkaline phosphatase, 1:100, Agilent Dako, Santa Clara, CA, USA). All sections were counterstained with hemalaun/permanent red solution (Agilent Dako) and examined by light microscopy (BX51, Olympus, Tokyo, Japan) using a ×40 objective (Plan N ×40/0.65 W, Olympus).

### Statistics

For evaluation of the alternative model, primary and secondary experimental outcomes were defined: primary outcomes were contraction of the perforation (mm^2^) and the epithelialization of the defect margin (yes/no). Secondary outcomes were neovascularization (intravital microscopy and immunohistochemistry) and inflammation infiltration (intravital microscopy and immunohistochemistry) to generate baseline data for future experiments. Statistical analysis was performed using repeated measures ANOVA/mixed effects analysis, followed by Tukey's or Sidak's multiple comparisons test (GraphPad Prism8©).

## Results

This study examined a new wound healing model for TMPs. Four modified DSC preparations were tested for feasibility, defect epithelialization, reduction in the perforation's diameter, and microvascular parameters. The SP without prior preparation of the panniculus carnosus muscle showed the best results: The preparation of SP was easy to perform. Cornified squamous epithelium overgrew the perforation's edge and the wound did not contract spontaneously. Meanwhile, the model enabled repetitive intravital visualization of the defect size and the microcirculation.

The preparation of the SP, SP-CS, and AP-CS were well tolerated and few adverse events occurred. In particular, preparation of the symmetric defects was straightforward, replicable, and did not require microsurgery. Four of 5 animals in the SP group survived for 20 days without adverse events, while 1/5 mice died during anesthesia on day 0. In the SP-CS group, late dropouts occurred on days 12, 16, and 20 because of known DSC complications (lateral tilting of the chamber, tear out of the sutures). Two of 5 animals survived for 20 days. Following preparation of the asymmetric preparation (with coverslip), 1/5 animals was lost to lateral chamber tilting on day 15, and 4/5 mice could be examined for 20 days. However, the AP led to wound contamination and dehydration: All animals were sacrificed because of chamber infection or necrosis before the first intravital microscopy on day 4. Therefore, testing of asymmetric preparations without coverslip was suspended.

**Fig. 4:**
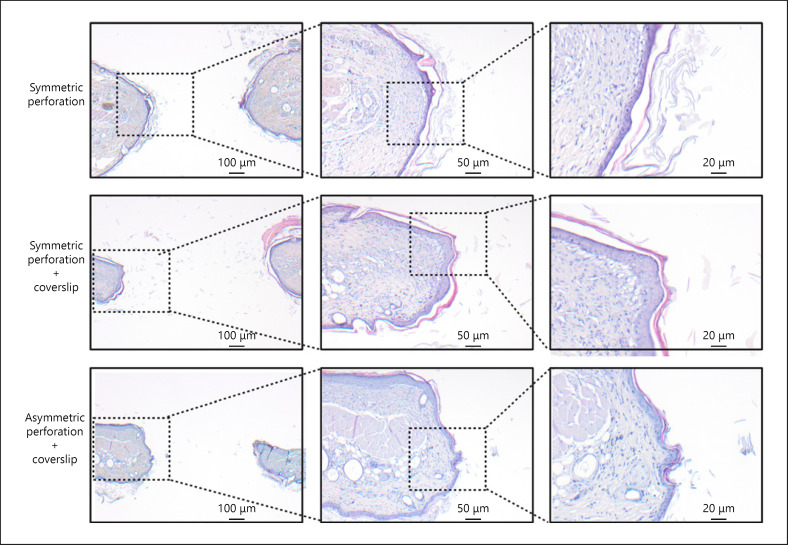
Premature epithelialization of the wound. HE staining of the DSC tissue was performed on day 20. The remaining defect is illustrated 20 days after preparation of a SP, SP-CS, and AS-CS (×5, ×10, ×20 from left to right). The edge of the defect was overgrown by squamous epithelium in all groups. Premature wound epithelialization without defect closure is characteristic for tympanic membrane defects. DSC, dorsal skinfold chamber; SP, symmetric perforation; SP-CS, symmetric perforation with coverslip; AP-CS, asymmetric perforation with coverslip.

Consistent with the main pathology of persisting TMPs, the edges of the defect epithelialized in all remaining preparations (shown in Fig. 4). Even after removal of 1 epithelial layer in asymmetric preparations (with coverslip), the remaining epithelial layer overgrew the defect. However, increased exposure of subcutaneous tissue and coverslip application induced detritus accumulation within the defect and impeded planimetry of the viable defect margins. In the preparations with coverslip, detritus accumulated within the perforation and covered larger areas in the perforation: 88.28 ± 6.60% in symmetric perforations with coverslip and even 96.93 ± 3.07% (average of days 4–20) in asymmetric perforations with coverslip. In symmetric perforations without coverslip, detritus secretion was lower and covered 51.84 ± 3.83% of the defect.

**Fig. 5:**
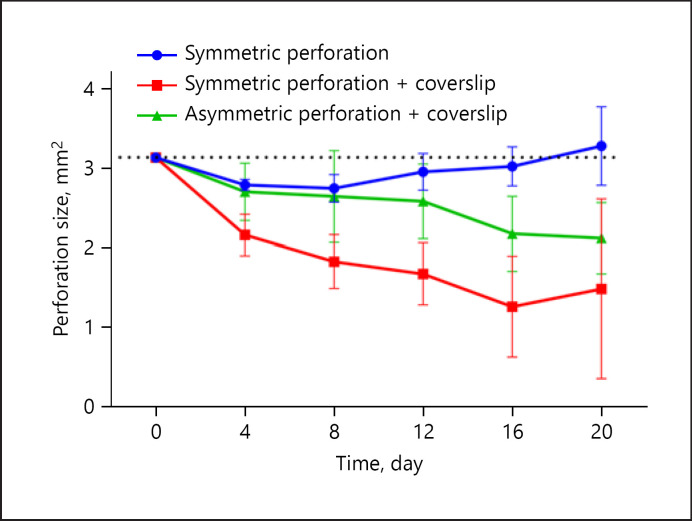
Repetitive planimetry of the perforation size. All defects were prepared using a 2-mm biopsy punch (Æ 3.14 mm^2^). Following preparation of a SP (*n* = 5, dropout 1/5 at day 0, blue) the defect size remains unchanged throughout 20 days. However, preparation of a SP-CS (*n* = 5, dropout 3/5 at days 12, 16, 20, red) or an AS-CS (*n* = 5, dropout 1/5 at day 15, green) increases spontaneous wound healing and contraction of the perforation. SE was lowest for the SP (without coverslip). Values are given as means ± SEM of independent experiments. SP, symmetric perforation; SP-CS, symmetric perforation with coverslip; AP-CS, asymmetric perforation with coverslip; SE, standard error.

Reduction of the perforation did not occur in symmetric perforations without coverslip (shown in Fig. 5). First, a defect with a size of 3.14 mm^2^ was punched and remained constant with 3.00 ± 0.23 mm^2^ on day 12 and 3.29 ± 0.50 on day 20 (*p* = 0.99 day 1 vs. last measurement). The application of a coverslip after preparation of a SP (SP-CS) led to reduction of the perforation starting on day 4 (2.17 ± 0.26 mm^2^); the resulting perforation contracted significantly to a size of 1.49 ± 1.13 mm^2^ on day 20 (*p* < 0.05, day 1 vs. last measurement). In the third group, AP-CS, intermediate spontaneous reduction of the perforation was observed. The defect size was stable and comparable to SP for 12 days (2.59 ± 0.47). However, during the third week, the defect contracted up to a final size of 2.15 ± 0.45 mm^2^ (*p* = 0.22, day 1 vs. last measurement).

Additionally to the constant defect size, the standard error/deviation of the measurements was lowest for the SP: SEMs were significantly lower for symmetric perforations without coverslip (0.24 ± 0.07 mm^2^) than symmetric perforations with coverslip (0.55 ± 0.16 mm^2^) and asymmetric perforations with coverslip (0.46 ± 0.03 mm^2^) (SP vs. SP-CS *p* < 0.05; SP vs. AP-CS *p* < 0.05; SP-CS vs. AP-CS *p* = 0.39). High SEMs were associated with detritus secretion, which impeded precise measurement of the perforation size.

**Fig. 6:**
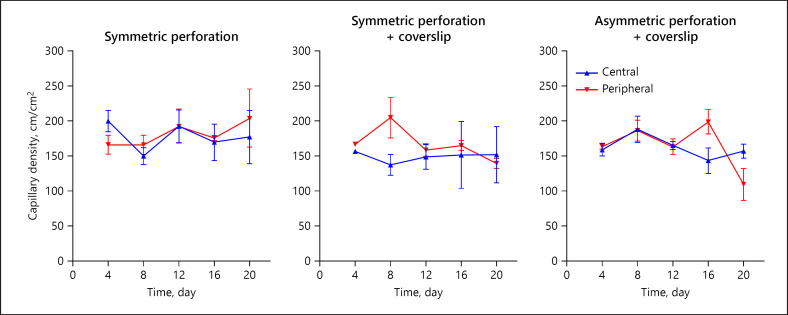
Repetitive analysis of functional capillary density. Functional capillary density (cm/cm^2^) was measured by intravital fluorescence microscopy following iv FITC-dextran injection. Two areas of interest were defined by the distance to the perforation: Central area = close to the perforation (within 1 field of view using ×20 magnification, blue) and peripheral area = distant to the perforation (>2 fields of view using ×20 magnification, red). Computer-guided image analysis was workable in all preparations (SP-CS, AP-CS). The functional capillary density was equal in areas close to the perforation and distant to the perforation. Comparison of central areas (underneath a wound dressing) and peripheral areas (beyond a wound dressing) enables for blood flow assessment depending on the wound therapy. Values are given as means ± SEM of independent experiments (*n* = 5, dropouts: symmetric perforation 1/5 at day 0; SP-CS 3/5 at days 12, 16, 20; AS-CS 1/5 at day 15). FITC, fluorescein isothiocyanate-labeled; SP, symmetric perforation; SP-CS, symmetric perforation with coverslip; AP-CS, asymmetric perforation with coverslip.

All DSC preparations enabled repetitive intravital microscopy and measurement of the capillary density. The capillary density was measured in defined central (close to the defect) and peripheral areas (shown in Fig. 6). The capillary density was consistent between central/peripheral, between the groups, and longitudinally throughout the course of the experiment (*p* > 0.05).

**Fig. 7:**
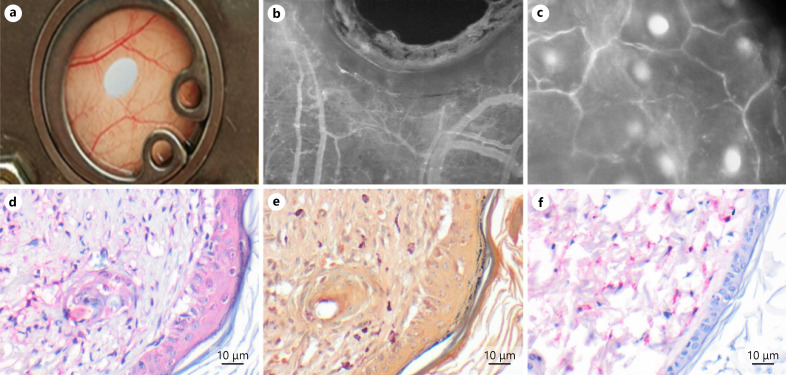
Representative images of the symmetric DSC perforation. **a** Macroscopic image of the perforation on day 0. **b** Intravital fluorescence microscopy (FITC-dextran) of the perforation and the surrounding vessels at day 8 (×50). **c** Intravital fluorescence microscopy (FITC-dextran) of a central capillary field at day 8 (×200). Visualization of wound healing by means of immunohistochemistry: Ki-67 − proliferation (**d**); CAE − leukocyte activation (**e**); F4/80 − macrophages (**f**). DSC, dorsal skinfold chamber; FITC, fluorescein isothiocyanate-labeled; CAE, chloroacetate esterase.

The intravital visualization of leukocyte migration required preparation of the panniculus carnosus muscle. Therefore, intravital measurement of the inflammation was possible for AP-CS (which in turn performed weak in spontaneous reduction of the perforation), but not in SP. Inflammation and proliferation were therefore measured by postmortem CAE, Ki-67, and F4/80 immunofluorescence microscopy (shown in Fig. 7). CAE-, Ki-67, and F4/80-positive staining was low, indicating low inflammation and proliferation. Cells were located subcutaneously, surrounding the vessels of the panniculus carnosus muscle. The regenerated epithelium and the subepidermal layers were negative for immune-infiltration and proliferation.

In summary, cornified squamous epithelium overgrew the edge of the perforation in all groups. Straightforward perforation without coverslip and without microsurgical preparation was favorable: spontaneous reduction of the perforation was marginal throughout 20 days and the neovascularization was visualized sufficiently for off-line analysis. Microsurgical preparation before biopsy punch perforation and sealing with a coverslip enabled repetitive high-quality intravital fluorescence microscopy. However, spontaneous reduction of the perforation occurred frequently following application of a coverslip.

## Discussion

Recent progress in tissue engineering brought up plenty of biomaterials for skin wound treatment as well as potential new therapies for TMPs. All potential scaffolds substantially differ in manufacturing processes (electrospinning and thermal inkjet printing), materials (gelatin and collagen PGS), physical characteristics (nanofiber alignment and porosity), and biological interactions (growth factors) [3, 26, 69].

Safe and effective clinical application requires systematic in vitro and in vivo evaluation of biocompatibility and effectivity [70]. Current orthotopic TMP models have 2 major limitations, which impede progress in tympanic membrane regeneration: Spontaneous defect closure without a reliable defect size and lack of wound visualization after biomaterial application. Here, analysis is based on (immuno-)histology of a variable defect after application of 1 isolated biomaterial sample at 1 single time point (Table 1). Here, we propose a modification of the DSC with standardized epithelialized defect as a model for the pathophysiology of TMP healing. In contrast to histological analysis, the proposed model enables repetitive intravital visualization and measurement of functional parameters [63].

The DSC full-thickness defect reflects key elements of tympanic membrane wound healing, such as lack of wound bed and scarce growth factor supply. We showed that cornified squamous epithelium overgrows the perforation's edge and that the DSC enables repetitive analysis of the defect size and angiogenesis after full-thickness perforation. Using established analysis procedures, the modified model provides an effective platform for the screening of bioactive materials and transferring progress in tissue engineering to the special conditions of tympanic membrane wound healing.

Premature defect epithelialization is a major factor in tympanic membrane wound healing and has to be addressed in any tympanic membrane model [2, 13, 18, 19, 20]. In the proposed model, an epithelial layer overgrew the wound within days after cold-steel injury, without further mechanical or chemical manipulation. Premature epithelialization was related to the small wound surface of 3.77 mm^2^. Epithelialization also occurs in rodent orthotopic models. However, contraction is the major factor in rodent wound healing and leads to high rates of spontaneous closure [71]. To prevent spontaneous closure, tympanic wound healing in rodent orthotopic models must be attenuated by thermal/laser myringotomy and topical drug application (which may interfere with the effects of test substances) (shown in Table 1). In human wound healing contraction is less important, while cell migration and proliferation are more pronounced [71]. In the proposed model, the fixed extension of the skin in the DSC minimizes unwanted wound contraction and pronounces wound healing by migration and proliferation [25, 72]. Likewise, the tympanic membrane stretches between the external meatus and the ossicles, while it lacks contractile elements (shown in online suppl. Fig. 2; see www.karger.com/doi/10.1159/000519774 for all online suppl. material). The full-thickness defect in the DSC can thus particularly simulate the dependence on proliferation and cell migration in tympanic membrane defects.

Among the 4 preparations tested, SP was most suitable: SP preparation was straightforward and required <10 min. Importantly, the perforation size remained constant throughout 20 days and planimetry was most replicable. SP also appears more natural because the human tympanic membrane defect is likewise surrounded by air on both sides.

On the contrary, application of a coverslip and sodium chloride led to moderate (AP-CS) or intense (SP-CS) reduction of the defect size. Additionally, more detritus remained after coverslip application (because of the closed chamber). In planimetry, the boundary of the vital tissue was used to determine the diameter and devitalized tissue (scab/detritus) was not considered for the measurements. However, detritus complicated precise identification of the vital wound margin. This is reflected in the higher standard deviations for the coverslip preparations. In future experiments, temporary coverslip application may decrease detritus and increase vital margin identification by high-quality intravital microscopy.

In SP and SP-CS, the remaining skin limited intravital microscopy quality to the measurement of the functional capillary density in the subcutaneous plexus. Arterioles and venules in a deeper layer were visible for morphological characterization and recovering of defined areas of interest. However, the intact layer of skin restricted functional measurements and leukocyte quantification. The AP-CS showed characteristic DSC intravital microscopy quality and enabled quantification of functional microvascular parameters and leukocyte visualization. Potentially interfering detritus was localized within the perforation (where major vessels were disrupted during preparation), but not in the microsurgically prepared area for intravital microscopy. Considering moderate spontaneous defect shrinkage and detritus deposition, AS-CS may be an alternative in special scientific issues, if the analysis of the microcirculation is the primary outcome and defect size is secondary.

However, defect size is the usual primary outcome in wound healing assays. Therefore, lower standard deviations in SP will be superior in these designs (e.g., 4 groups of *n* = 8; for a relevant difference of 20%, *F*-test -ANOVA).

Intravital microscopy measurements of the capillary density showed no differences between the groups (SP, SP-CS, and AP-CS) and between central and peripheral areas. In contrast, previous DSC studies showed characteristic microvascular changes (formation of circular and radial vessels) at the wound boundary [62]. In this preparation, the wound was not punched through both layers of the dorsal skinfold. Therefore, a wound bed was available to provide sufficient cytokines and wound healing could pass through the physiological phases of inflammation and proliferation. The proposed DSC preparation has a different pathology: the wound bed is limited to the small lateral surface of the perforation and the wound essentially lacks support for cell migration and cytokine supply. Scarce cytokine supply shortens the proliferation and inflammation phase of wound healing. Both are essential for the characteristic microvascular changes of physiological wound healing. Low microvascular wound healing activity is supported by low CAE/Ki-67 expression in immunohistology.

The AP and SP-CS preparations were not suitable as TMP models. The AP was prone to contamination, infection, and necrosis because of the exposed fascia and microvessels of the panniculus carnosus muscle. SP-CS was inferior to AP-CS regarding intravital microscopy and to SP regarding spontaneous reduction of the perforation. However, improved defect reduction after coverslip and sodium chloride application may point to the potential of moist wound healing for epithelialized defects. Higher epithelium in the SP-CS group may be a correlate of improved wound healing (shown in Fig. 4). Likewise in humans, a positive correlation of moist and bloody tympanic membrane defects with spontaneous closure has been described for human TMPs [13, 73].

The primary advantages of the DSC full-thickness preparation are the repetitive examination of the wound by planimetry and intravital microscopy in a well-established setup [25, 47, 62, 63, 72, 74, 75]. Intravital and confocal microscopy using fluorescein isothiocyanate-labeled-dextran, Rhodamine 6G, or autofluorescent mice (red tomato) are accepted standards in wound healing research [47]. In the present study, SKH1 hairless mice were used to avoid shaving and minimize anesthesia time. In general, any transgenic mouse species is suitable for DSC experiments. On the contrary, most orthotopic models are limited to wild-type animals. Even though mice are smaller than the animals in orthotopic models, the 10-mm diameter of the DSC corresponds to the human tympanic membrane [76]. The defect size is variable depending on the skin biopsy punch. We prepared the common defect size of human TMPs(2–4 mm^2^) using a 2-mm punch (= 3.14 mm^2^) [77]. The perforation is easily accessible and enables a repetitive change of wound dressings. Potential tympanic membrane wound dressings cannot be fixed by suturing and must remain by adhesion. In the DSC, the wound dressing can be fixed under a coverslip or grid insert to avoid manipulation by the animal (shown in online suppl. Fig. 1).

However, the DSC full-thickness defect has several limitations as a tympanic membrane model. The microarchitecture of the respective tissues differs markedly (shown in online suppl. Fig. 2): The tympanic membrane is extraordinarily thin to ensure good sound transmission (100 μm). The double skinfold has a diameter of up to 600 μm. The wound surface area of a circular perforation correlates with the thickness and the diameter (lateral surface of a cylinder). For a 2-mm defect diameter, the tympanic membrane wound surface measures 0.63 mm^2^ and the DSC wound surface 3.77 mm^2^. Even though the larger wound surface in the DSC model may provide better support for proliferation and defect closure, HE and immunohistology have shown the same pathology of inhibited proliferation and premature epithelialization.

Histologically, the DSC comprises 2 corresponding skin layers with a well-vascularized lamina propria and panniculus carnosus muscle. The main wound healing mechanism in the DSC is proliferation and migration of squamous epithelial and subepithelial cells. The tympanic membrane carries low cornified squamous epithelium on the external auditory meatus' side and respiratory mucosa on the middle ear's side. Both layers are separated by 2 layers of collagen fibers (radial/circular) and the respective lamina propria (with low vascularization). The respiratory epithelium of the tympanic membrane has a minor role in epithelialization because proliferation is lower than the squamous epithelium [78, 79, 80]. Therefore, squamous epithelium overgrows the perforation's edge and ends closure of the TMP. In the dorsal skinfold chamber, epithelialization − without closure of the perforation − occurs comparably, despite the larger wound area and the favorable wound. The DSC full-thickness perforation may therefore model the main pathology of the persisting TMP: The imbalance between the size of the perforation and the size of the actual exposure of subepithelial tissue.

Another obvious limitation is the heterotopic location: the intact tympanic membrane separates the middle ear from the external auditory meatus. Both have a specific microclimate: the tympanic membrane has a temperature of ∼37.3°C (core body temperature) and the humidity in the human ear canal is 50% (±10%) [81]. In comparison, the skin temperature of the mouse is lower at 33°C, while the relative humidity of 40–65% is the same in the laboratory animal husbandry. However, after application of a wound dressing on the lateral surface of the tympanic membrane, the wound is only exposed to the moist and warm air of the middle ear. This may be beneficial to wound healing compared to the open chamber preparation (SP). Even though air humidity and temperature play subordinate roles in wound healing, the absence of the specific microclimate of the ear remains a limitation of the heterotopic model.

Anyway, the DSC has always been a heterotopic model for wound healing, thrombogenesis, cancer treatment, and biomaterial integration. In this artificial environment standardized, repetitive, and intravital visualization of pathophysiology has provided valuable results [47, 62, 63].

Another limitation of the DSC is the restriction to maximum 21 days of examination. The DSC tilts laterally between 10 and 21 days. Tilting causes animal discomfort and bending of the entering vessels with decreased blood supply. Orthotopic tympanic membrane models suffer high rates of spontaneous closure but enable long-term observation after establishment of the perforation. However, the DSC observation period covers the inflammation-proliferation transition of wound healing. This transition is critical in wound healing, requires specific cell-mediator interactions, and determines the long-term fate of the wound [26, 82].

Finally, a moderate distress for the animals must be considered in all DSC experiments (surgery, physical burden, and repetitive anesthesia). In this study, the dropouts were comparatively high. In SP and AP-CS, 1 of 5 animals dropped out before day 20 (anesthesia complication on day 0 and >90° chamber tilting on day 15. The observation time of 20 days after chamber preparation is relatively long compared with previous experiments and a dropout of 10–20% must therefore be expected. Chamber tilting in the second and third week, which was responsible for most of the dropouts, can be minimized significantly by light-weight chambers in future experiments [82].

## Conclusion

In the field of TMPs, orthotopic models have made progress in controlling spontaneous closure but essentially lack repetitive visualization of the wound-biomaterial interface. Therefore, a preclinical model that enables standardized screening of promising biomaterials is needed. The DSC with a full-thickness defect represents a valuable tool to optimize scaffolds with sequential growth factor release and mitigate premature epithelialization in tympanic membrane wound healing. The presented model may therefore become a standard model to transfer progress in tissue engineering to tympanic membrane regeneration. Subsequently, effective biomaterials can be evaluated specifically in orthotopic models and in clinical studies. Future standardized development of effective tympanic membrane wound dressings may prevent chronic perforations and microscopic myringoplasty for selected patients.

## Statement of Ethics

All animal experiments were approved by the local governmental authority: Landesamt für Landwirtschaft, Lebensmittelsicherheit, and Fischerei Mecklenburg-Vorpommern (7221.3-1-033/19), in accordance with the governmental animal protection law and the EU Guideline 2010/63/EU.

## Conflict of Interest Statement

The authors declare that the research was conducted in the absence of any commercial or financial relationships that could be construed as a potential conflict of interest.

## Funding Sources

The study was supported by a grant from the Rostock University Medical Center in the framework of the FORUN program 2020.

## Author Contributions

All authors contributed to the study, conception, and design. D.S. and E.G. analyzed and interpreted the data. D.S. wrote the manuscript. D.S. and C.L. performed all experiments. S.v.B., S.S., R.M., and B.V. participated in manuscript finalization and critically revised the manuscript. D.S., E.G. and B.V. designed the study and participated in the writing of the manuscript. All authors read and approved the final manuscript.

## Data Availability Statement

The datasets generated for this study are available on request to the corresponding author.

## Supplementary Material

Supplementary data

## Figures and Tables

**Table 1:** Recent orthotopic models, variable spontaneous closure rates, and methods for inhibition of wound closure [3, 9, 27, 31, 32, 34–36, 40, 41, 45, 83–94]

Author	Year	Spontaneous closure, %	Inhibition of wound closure	Perforation method	Animal species	*N*	Observation time, weeks	Experimental treatment
Dirain et al. [41]	2018	100	Coagulation	Laser	Rat	9	4	Antibiotic ear drops

Emami et al. [36]	2014	100	Coagulation + epithelialized flap	Thermal myringotomy + infolding	Chinchilla	8	6	M&M

Kaftan et al. [35]	2012	100	None	Myringotomy	Rat	6	4	M&M

Shen et al. [83]	2013	100	None	Myringotomy	Rat	30	4	Silk/fibrin, collagen, paper, gelfoam

McFeely et al. [34]	2000	90	Epithelialized flap	Myringotomy + infolding	Chinchilla	10	4	Alloderm

Ramalho et al. [84]	2006	45	Coagulation + epithelialized flap	Thermal myringotomy + infolding	Chinchilla	10	4	EGF, pentoxifylline

Jang et al. [9]	2013	43	Topical drugs + coagulation	Thermal myringotomy + mitomycin + dexamethasone	Guinea pig	10	6	3D collagen scaffold

Downey et al. [85]	2003	35	Coagulation + epithelialized flap	Thermal myringotomy + infolding	Chinchilla	17	10	Alloderm

Wieland et al. [86]	2010	31	Coagulation + epithelialized flap	Thermal myringotomy + infolding	Chinchilla	11	8	Polyglycerol sebacate-engineered plugs

Kuo et al. [3]	2018	25	None	Myringotomy	Chinchilla	4–6	21	Bioprinted gelatin

Wang et al. [45]	2017	20	Topical drugs	Myringotomy + mitomycin + dexamethasone +Rat ventilation tube	40	10	M&M

Seonwoo et al. [27]	2013	20	Topical drugs + coagulation	Thermal myringotomy + mitomycin + dexamethasone	Rat	24	10	EGF chitosan patch

Laidlaw et al. [87]	2001	15	Coagulation	Thermal myringotomy	Chinchilla	23	8	Alloderm

Langsten et al. [40]	2019	13	Topical drugs	Myringotomy + myringotomy + mitomycin + dexamethasone	Mouse	5–50	8	M&M

Seonwoo et al. [31]	2019	12	Coagulation	Thermal myringotomy	Rat	38	8	EGF-releasing nanofiber patch

Santa Maria et al. [88]	2011	12	Topical drugs	Myringotomy + metalloproteinases, Eustachian Rat tube obstruction	18	12	M&M

Deng et al. [89]	2009	11	Epithelialized flap	Myringotomy + infolding	Guinea pig	40	8	Acellular dermis and dura mater

Rahman et al. [90]	2008	10	Coagulation	Laser	Rat	10	4	Stem cells

Spiegel et al. [91]	2005	0	Epithelialized flap	Myringotomy + infolding	Chinchilla	10	8	Acellular porcine submucosa

Parekh et al. [92]	2009	0	Epithelialized flap	Myringotomy + infolding	Chinchilla	11	8	Urinary bladder matrix

Weber et al. [93]	2009	n.a.	Coagulation	Thermal myringotomy	Chinchilla	11	6	Alginate

Sundback et al. [94]	2012	n.a.	Coagulation + epithelialized flap	Thermal myringotomy + infolding	Chinchilla	11	8	Polyglycerol sebacate-engineered plugs

Aksoy et al. [32]	2018	n.a.	None	Myringotomy	Rat	10	3	PRP-soaked fat graft

Table 1 illustrates recent work on orthotopic tympanic membrane models and the variable spontaneous defect closure rates. Various perforation methods and animals have been tested to minimize spontaneous closure.
