# Supplementary material for: The Dorsal Skinfold Chamber as a New Tympanic Membrane Wound Healing Model: Intravital Insights into the Pathophysiology of Epithelialized Wounds
Source: Eur Surg Res. 2021 Dec 2;63(4):335–49. doi: 10.1159/000519774 (PMC9808650; doi:10.1159/000519774)
Supplement: Supplementary file 1 — Supplementary data [file esr-0063-0335-s01.docx]

**
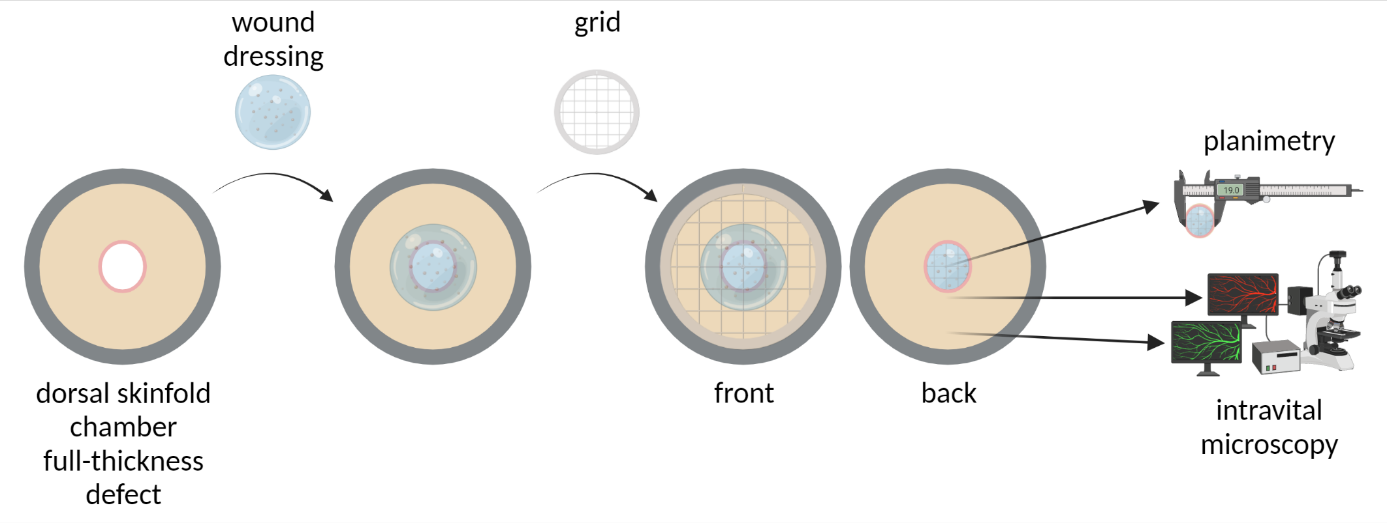
**

**Suppl. Fig. 1: Wound dressing application and microscopy.** Wound dressings are applied on the front side of the perforation. Animal manipulation may be prevented by application of a grid or a coverslip. After biomaterial application, the wound margins remain accessible for planimetry and intravital microscopy from the back side. Intravital microscopy can be performed in central areas (under the influence of the biomaterial, red) and in peripheral areas (outside of the biomaterial, green).


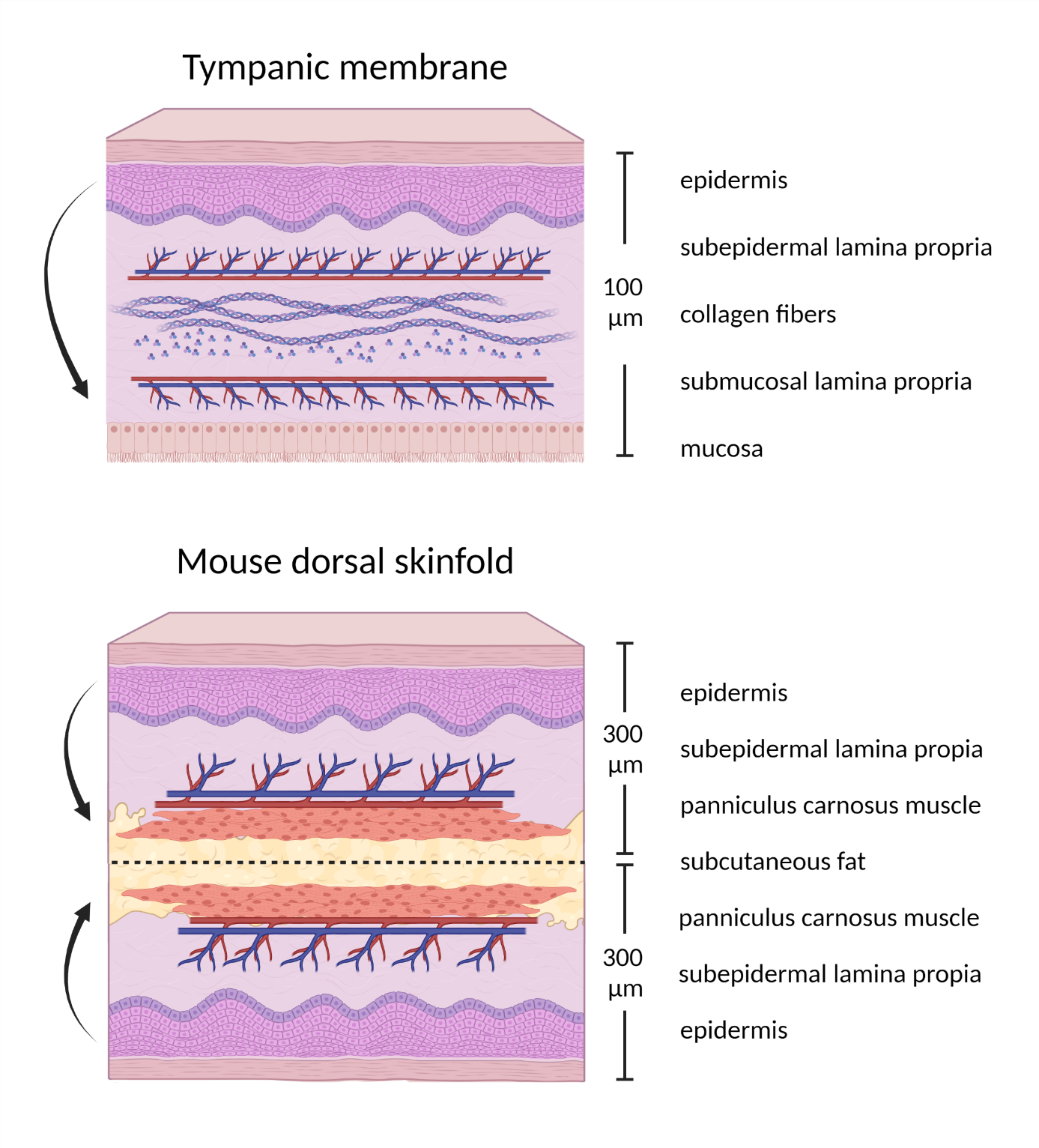


**Suppl. Fig. 2: Tympanic membrane and mouse dorsal skinfold chamber microstructure.** Both tissues comprise two epithelial layers separated by vascularized connective tissue. The key differences are the respiratory mucosa, the small diameter, and the absence of a muscular layer in the tympanic membrane. However, following full-thickness perforation both tissues share the wound healing mechanism of epithelialization by epidermal squamous epithelium (indicated by arrows).
